# Hyperspectral Imaging and Spectrometry-Derived Spectral Features for Bitter Pit Detection in Storage Apples

**DOI:** 10.3390/s18051561

**Published:** 2018-05-15

**Authors:** Sanaz Jarolmasjed, Lav R. Khot, Sindhuja Sankaran

**Affiliations:** 1 Department of Biological Systems Engineering, Washington State University, PO Box 646120, Pullman, WA 99164, USA; s.jarolmasjed@wsu.com (S.J.); lav.khot@wsu.com (L.R.K.); 2 Center for Precision and Automated Agricultural Systems, Washington State University, 24106 North Bunn Road, Prosser, WA 99350, USA

**Keywords:** Honeycrisp, visible–near-infrared spectroscopy, image processing, classification

## Abstract

Bitter pit is one of the most important disorders in apples. Some of the fresh market apple varieties are susceptible to bitter pit disorder. In this study, visible–near-infrared spectrometry-based reflectance spectral data (350–2500 nm) were acquired from 2014, 2015 and 2016 harvest produce after 63 days of storage at 5 °C. Selected spectral features from 2014 season were used to classify the healthy and bitter pit samples from three years. In addition, these spectral features were also validated using hyperspectral imagery data collected on 2016 harvest produce after storage in a commercial storage facility for 5 months. The hyperspectral images were captured from either sides of apples in the range of 550–1700 nm. These images were analyzed to extract additional set of spectral features that were effective in bitter pit detection. Based on these features, an automated spatial data analysis algorithm was developed to detect bitter pit points. The pit area was extracted, and logistic regression was used to define the categorizing threshold. This method was able to classify the healthy and bitter pit apples with an accuracy of 85%. Finally, hyperspectral imagery derived spectral features were re-evaluated on the visible–near-infrared reflectance data acquired with spectrometer. The pertinent partial least square regression classification accuracies were in the range of 90–100%. Overall, the study identified salient spectral features based on both hyperspectral spectrometry and imaging techniques that can be used to develop a sensing solution to sort the fruit on the packaging lines.

## 1. Introduction

Bitter pit is a physiological disorder in fruits such as apples and pears that develops before harvest and during storage. Bitter pit produces dark-brown, corky and roundish lesions, which appear and grow on the skin or in the flesh of the fruit. The bitter pits are prominent in the calyx end of the fruit [1,2]. Bitter pit was recognized as an important disorder when it resulted in severe economic issues during export and storage [2]. This disorder reduces marketability and product utilization value and causes significant post-harvest losses in apples [3]. Bitter pit occurs in certain varieties of apple cultivars and some research studies have reported 30% loss in Honeycrisp apples caused by bitter pit [4].

Miqueloto et al. [4], do Amarante et al. [5], and de Freitas et al. [6] suggested that bitter pit occurrence is related to the ratios of mineral nutrients such as calcium, magnesium and potassium in apples. These studies indicate that fruits with low calcium concentration, where the calcium is partially replaced by magnesium in calcium compounds, result in apple bitter pit formation. Calcium contributes to the membrane structure, but magnesium cannot satisfy a similar role in support as calcium does in the cell structure. Without enough cell support, the plasma membrane structure can be weakened, which can lead to cell death [6] and the collapsed cells appear as brown spots, known as bitter pit, on the fruit.

Sensing techniques have been used in food-quality inspection such as fluorescence [7,8,9,10], X-ray imaging [11,12,13], near-infrared spectroscopy [14,15,16,17], electronic nose [18,19], electronic tongue [20,21], thermal imaging [22,23], and hyperspectral imaging [24,25,26]. Fluorescence sensing [7,8], near-infrared spectroscopy [14,15], and computed tomography imaging [11] have been used in particular for apple bitter pit detection. Over the past few years, hyperspectral imaging has developed as a more reliable tool to inspect food quality [27,28,29,30,31] because of the spatial as well as the spectral information that it provides. Each pixel in the hyperspectral image contains the full spectrum that can be used to characterize that particular pixel [32]. Reflectance hyperspectral imaging in the range of visible to near-infrared measures the reflectance of the tissue beyond the visible spectrum. The spectral profile alters based on the changes in the chemical and physical properties [33], thus serving as a valuable tool for characterizing the disorder on the fruit. In this study, we investigate hyperspectral imaging for detecting bitter pit in apples.

In our previous studies [14,15], spectral features were selected from visible–near-infrared spectral reflectance data (350–2500 nm) that could be used to detect bitter pit presence with the high accuracy. In this study, the overall goal was to validate these extracted spectral features using hyperspectral imaging systems (550–1700 nm). The reason for this imaging-based validation is that the multispectral and hyperspectral imaging systems offer pixelated spectral information, which could be integrated with fruit sorting units for identification of bitter pit apples before storage.

## 2. Materials and Methods

### 2.1. Samples 

Table 1 shows the details on harvested apple samples from 2014 to 2016 as well as the fruits collected from the storage facility (2016 harvest, data collected in 2017). In the 2014, 2015 and 2016 harvest seasons, healthy and bitter pit Honeycrisp apple samples were harvested and stored in a controlled environment at 5 °C for 63 days to avoid samples appearing to be healthy that may develop bitter pit during storage. The visible–near-infrared spectral reflectance data (350–2500 nm) were collected from these samples. In addition, Honeycrisp apple samples were collected from Borton Fruit storage facility in February 2017. The samples were kept in the commercial storage condition from the harvest season in September 2016. In the apple harvest season, bitter pit apples were not picked, and apples that developed bitter pit during approximately five months of storage were visually selected for this study. The visual selection (independent of severity) allowed data collection with balanced sample size. The samples were transported to the Center for Precision and Automated Agricultural Systems (CPASS) in Prosser, WA, USA, in boxes for visible–near-infrared spectral data (350–2500 nm) and image (550–1700 nm) acquisition. Bitter pit apples were separated from the healthy ones based on two harvest locations in the facility. 

### 2.2. Visible–Near-Infrared Spectra Acquisition and Analysis

The spectral reflectance of each set of apples was acquired using a spectroradiometer (Spectra Vista Corporation, SVC HR-1024i, Poughkeepsie, NY, USA). The fiber optic probe was used to collect spectra from three sides of the apple around the stem-calyx axis. The captured spectra ranged between 350–2500 nm and were radiometrically calibrated using the reference panel (Spectralon Reflectance Target, Labsphere^®^, North Sutton, NH, USA) mounted on the reflectance probe. The probe field of view covered the area of a circle with a diameter of 12 mm.

The captured spectra from the 2014 season were used to select spectral features to identify bitter pit [14,15] based on partial least square regression (PLSR), stepwise discriminant analysis (SDA), stepwise regression analysis, and rank features technique. Using some of these spectral features, in addition to water absorption, -OH bond, and red edge spectral features, the final set of spectral features were determined as 730, 980, 1135, 1250, and 1450 nm, which were evaluated on 2014, 2015, 2016, and 2017 (2016 after storage) datasets. For this purpose, the classification accuracy of the datasets with the selected features was estimated using PLSR and k-nearest neighbors (KNN). PLSR is used to construct predictive models in case of a considerable number of features. It reduces the predictors to a smaller number of components and applies least square regression on these uncorrelated components [34]. KNN classifies based on the majority vote of its nearest neighbors in the feature space. The closer neighbors get higher weight and the class is assigned according to the most common class among its KNN [35]. Later, these spectral features were also evaluated using images from the hyperspectral imaging system. 

### 2.3. Hyperspectral Image Acquisition

Data collection using a hyperspectral camera (Hyperspec Extended VNIR, Headwall Photonics Inc., Fitchburg, MA, USA) was performed after the fruit temperature was brought to room temperature. This line-scan camera has a spectral and spatial resolution of 12 nm and 320 pixels, respectively. The wavelength ranged between 550–1700 nm with 148 channels. Images were captured from the samples along the stem–calyx axis. Each apple was set individually on a black surface on the moving platform. The illumination source (halogen tungsten) was fixed on two sides of the fruit at 45° from the vertical axis to prevent shadows resulting from the shape of the fruit. The apples were placed on a small round base holder for stability during imaging, and the holder was not visible in the image. Images were captured from two sides of the sample with 180° of the apple central vector. For optimizing illumination without specular reflectance spots, different halogen tungsten light sources and light diffusers were evaluated. This noise from light sources on the surface of the sample has been reported in literature [36,37,38,39]. Although the light diffusers were successful in eliminating saturated (specular reflectance) points, the light intensity for reflectance capture was reduced considerably. Keresztes et al. [28] recommended elimination of such spots during image processing as the most efficient way for removing such noise. The images were acquired by the Hyperspec III^®^ software (Headwall Photonics Inc., Fitchburg, MA, USA). Before imaging, dark and white references were collected and the reflectance was calibrated internally. Later, these images were further processed in Matlab^®^ (Mathworks, Natick, MA, USA) for image analysis.

#### 2.3.1. Hyperspectral Image Processing 

**Figure 1:**
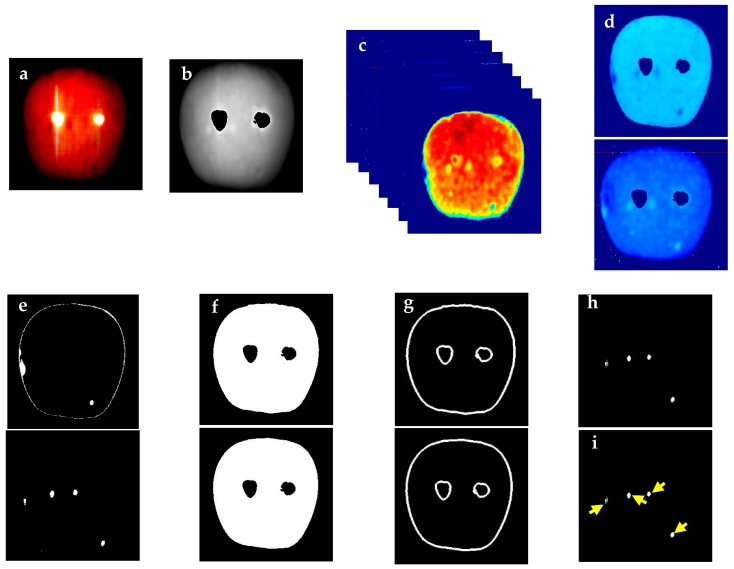
Automated hyperspectral image-processing algorithm process flow to detect area of bitter pitted spots: (**a**) normalized spectral value of each image pixel; (**b**) extracted mean spectral image (MSI) with elimination of saturation points; (**c**) extraction of spectral image features with potential for bitter pit detection; (**d**) above and below images generated by summing 665, 731 and 797 nm images (<1000 nm), and by summing 1217, 1283, 1349 and 1410 nm images (>1000 nm), respectively; (**e**) thresholding of corresponding above and below summed images individually; (**f**) noise removal, binarization, and masking with corresponding MSI image of corresponding above and below images; (**g**) detection and edge removal corresponding to above and below images; (**h**) addition of both processed summed images (above and below images shown in g); and (**i**) computation of number of bitter pits.

Image datasets were analyzed using a custom algorithm developed in Matlab image-processing toolbox (Figure 1). The batch of data was loaded into the software, and the mean spectral image (MSI) was extracted. MSI was used to eliminate specular reflection spots from each apple image (Figure 1b). The saturated spots showed 3–12 times higher reflectance than apple tissue depending on the wavelength range. This property was used to define a threshold range larger or equal to three times the reflectance of MSI and the resulting image was used to mask hyperspectral images. After thresholding to eliminate the saturated spots, each of 240 hypercubes (2 images per sample) was normalized using Equation (1).

(1) $$R_{n(i)}=\frac{R_{i}}{\sqrt{{(R}_{i}^{2}{+R}_{i+1}^{2}{+\ldots +R}_{n}^{2})}}$$

R_n(i)_ is the normalized reflectance for each wavelength, R_i_ is the reflectance of the same wavelength in the captured spectra, and n is the number of features within the spectral acquisition range. The images of the selected spectral features (730, 980, 1135, 1250 and 1405 nm) from visible–near-infrared spectral reflectance analysis were extracted. However, only 730, 1250 and 1410 nm spectral reflectance images were able to identify the bitter pit locations on the fruit with minimal noise. Therefore, all the spectral images acquired from the hyperspectral system were re-examined to select the best spectral features to classify apple healthy tissue and bitter pit.

The ranges showing the best discrimination between healthy and bitter pit tissues were determined. The selected wavelengths were in the ranges of 665–797 nm and 1217–1410 nm. The new set of the selected image features from hyperspectral image data were 665, 731, 797, 1217, 1283, 1349, and 1410 nm (Figure 1c). As each of the wavelength ranges showed slightly different defects on the fruit, one separate image was created for each range by averaging the reflectance spectra in that range (Figure 1d), and a threshold was set to detect the bitter pit spots (Figure 1e). For image processing, the images were transformed into a grayscale binary image. In this way, the white pixels represented the healthy portion, while the black pixels represented bitter pits. Filtering was applied to remove noise from the images. Morphological operations were used to enhance image segmentation and the MSI image was masked on the spectral image (Figure 1f). Due to the curvature at the edge of the fruit, the spectral reflectance intensities at different wavelengths were lower compared to the tissue. As this could lead to noisy data that may have features similar to bitter pit, an edge detection operation was applied to the images, and edges (border pixels) were removed from each image (Figure 1g). Finally, the two images were binarized and the thresholded image was masked on it for background removal. The resulting image only contained bitter pit spots (Figure 1h). In the absence of bitter pit, the algorithm reported the result as zero. In the presence of bitter pit, these points were calculated, and the number and area of bitter pits were reported (Figure 1i). All the processes of the algorithm were operated automatically on each batch of the dataset. The processing time for each image with the spatial resolution of the line scan camera was approximately 20 s. Parallel computing and multispectral imaging with specific wavelengths can reduce both of the processing and imaging time for applications in the packaging facility.

#### 2.3.2. Logistic Regression

**Figure 2:**
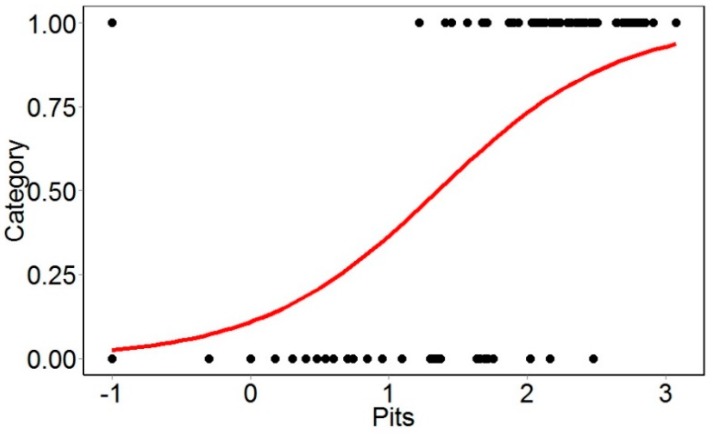
Logistic regression (logarithmic) to define the threshold of the pit area between healthy and bitter pit apples using the images.

Logistic regression was used to predict the threshold of the disorder in apples. Logistic regression is a statistical method that predicts the probability of each sample falling in either of the binary classes (healthy or bitter pit). It serves as a probability model to determine the threshold to classify the categorical data. This method is described thoroughly in Jarolmasjed et al. [11]. It is possible to estimate the optimized threshold for classification using this method. The threshold of the pit area is extracted from the model when the odds of the fruit being bitter pitted is 50%. This threshold is logarithmic in the model and equals 22.38 pixels when converted (Figure 2). This threshold was used as a feature to classify bitter pit and healthy apples. The distribution of the bitter pit area on apples was analyzed to categorize the apples in severely, moderately, and mildly affected fruit. Logistic regression was applied to the data from 665–797 nm and 1217–1410 nm separately, and the thresholds were determined as 5.10 and 1.99, respectively. 

#### 2.3.3. Re-evaluation of the Spectral Features in Visible–Near-Infrared Reflectance Spectral Data

The spectral reflectance data of the selected wavelengths extracted from hyperspectral images were re-analyzed using visible–near-infrared spectral reflectance data with PLSR and KNN. The classification methods were applied to the spectral reflectance data that were separated into training and testing datasets (3:1). The classifier performance was evaluated by randomizing the datasets three times. The algorithms were developed in Matlab software using the statistics and machine learning toolbox, and cross-validation of 5 was applied in each run during model development.

### 2.4. Statistical Analysis

Logistic regression and all statistical analysis were performed using R Studio (ver. 0.99.451, R Studio Inc., Boston, MA, USA). Results were inferred at 5% level of significance.

## 3. Results and Discussion

### 3.1. Classification using Selected Visible—Near-Infrared Reflectance Spectral Features

**Figure 3:**
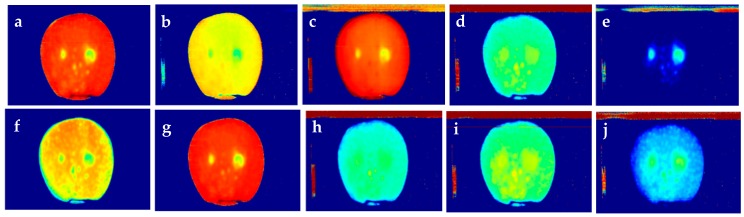
Hyperspectral images extracted from the selected visible–near-infrared reflectance spectral features: (**a**) 730 nm; (**b**) 980 nm; (**c**) 1130 nm; (**d**) 1250 nm; and (**e**) 1410 nm, and from the selected hyperspectral imaging features: (**f**) 665 nm; (**g**) 797 nm; (**h**) 1217 nm; (**i**) 1283 nm; and (**j**) 1349 nm in addition to 730 and 1410 nm.

Table 2 shows the classification accuracies of all four datasets using the selected visible–near-infrared spectral features (730, 980, 1135, 1250 and 1405 nm). These spectral features could classify visible–near-infrared reflectance data with an overall accuracy of 75–97%. The images associated with these spectral features were extracted from the hyperspectral data. However, except for 730, 1250 and 1410 nm, other images were unable to provide distinctions of bitter pit locations in the spatial level (Figure 3).

The hyperspectral images were examined to select spectral features capable of discriminating healthy and bitter pit tissues, and re-evaluated with visible–near-infrared spectral reflectance data. The selected features were 665, 731, 797, 1217, 1283, 1349, and 1410 nm. PLSR and KNN were applied to these features extracted from the visible–near-infrared spectral reflectance data (Table 2). Bitter pit creates internal breakdown of the flesh below the skin, and later it appears on the skin. Therefore using the near-infrared range of imaging can help in detecting the pits below the skin as it penetrates into the fruit’s flesh [40,41]. The fruit skin reduces light penetration into the fruit [42], and depending on the wavelength, the penetration depth can be different [40]. Lammertyn et al. [40] concluded that near-infrared light can penetrate 2–4 mm in Jonagold apple tissue in the range of 500–1900 nm. Their results showed that the depth is higher between 700–900 nm and lower at the chlorophyll absorption peak (692 nm) and water absorption band (1490 nm). With this information, it is possible to postulate that the wavelengths can potentially detect pre-symptomatic bitter pit in the subsurface of the fruit.

Literature also suggests that these wavelengths could be indicators of other fruit properties. Yang et al. [43] reported 665 and 793 nm as a couple of the most effective wavelengths that define blueberry fruit maturity. Yang et al. [44] described a steep rise between 640 and 683 nm that could describe the presence of frass on mature tomato. Barbin et al. [45] also observed efficient regression models of total viable count at 1344 and 1211 nm. They also concluded that 1211, 1241 and 1388 nm are the best features for psychrotrophic plate count in meat microbial contamination. In similar studies [46,47], the chosen wavelength for total viable counts in chicken breast included 1218 and 1345 nm. The discussed bands chosen in different research studies are in the close neighborhood of the bands selected in this study. Overall, the features of the hyperspectral image performed better in the PLSR classification of visible–near-infrared data specifically in reducing false positive.

### 3.2. Hyperspectral Image Data 

#### 3.2.1. Classification Results Based on Bitter Pit Area

The bitter pit area was used to classify the apples into two groups of healthy and bitter pit. The threshold was determined by logistic regression for using the entire selected wavelength as well as using 3 features and 4 features. The classification results are shown in Table 3. Although using a lower number of features does not significantly affect the accuracy, results indicate that using 7 features is effective in reducing the false positive that can prevent healthy apples being identified as bitter pit. Misclassification due to false positives may have occurred because of the slight defects on the healthy apple skin. To eliminate this error, future work can identify the size of each pit and remove them if smaller than a certain pixel number. Nicolai et al. [31] also showed that bitter pit was detectable using near-infrared hyperspectral imaging even without visible symptoms on the surface.

#### 3.2.2 Bitter Pit Area Distribution in Healthy and Bitter Pit Apples

**Figure 4:**
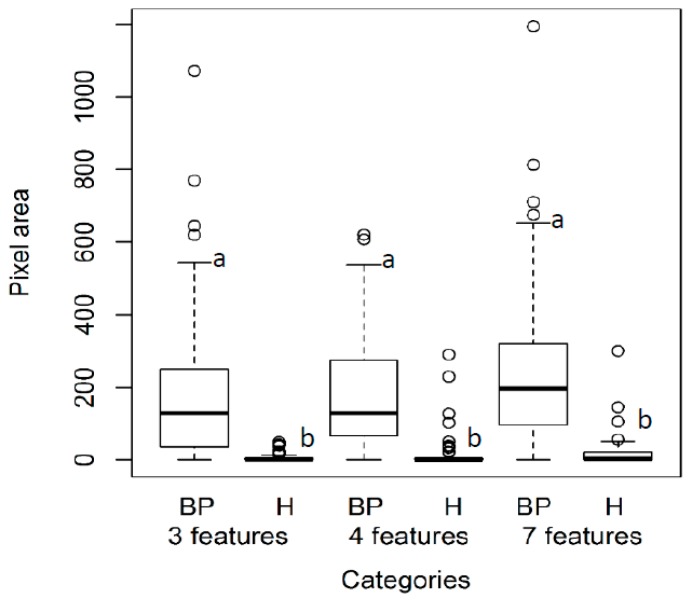
Bit distribution on healthy and bitter pit apples using the selected features. Three features refer to 665, 731, 797 nm, and four features refer to 1217, 1283, 1349, and 1410 nm. The least significant difference (LSD) test was conducted (α = 0.05) for each treatment, the same letter within each dataset shows treatments that were not significantly different.

The bitter pit area was useful in assessing the apple samples and distinguishing the bitter pit on the fruit surface. However, some bitter pit apples showed such a low bitter pit occurrence that it could be compared to selected anomalies on healthy apples. Figure 4 shows the distribution of the bitter pit area on apples when all of the features, as well as 3 and 4 features, were used. When all 7 selected features were used, the area was larger compared to the other two regions. This suggests that each of the 3 and 4 features are contributing to the selection of the bitter pit area. This result showed that using all 7 features provides better detection, although not significantly different. 

**Figure 5:**
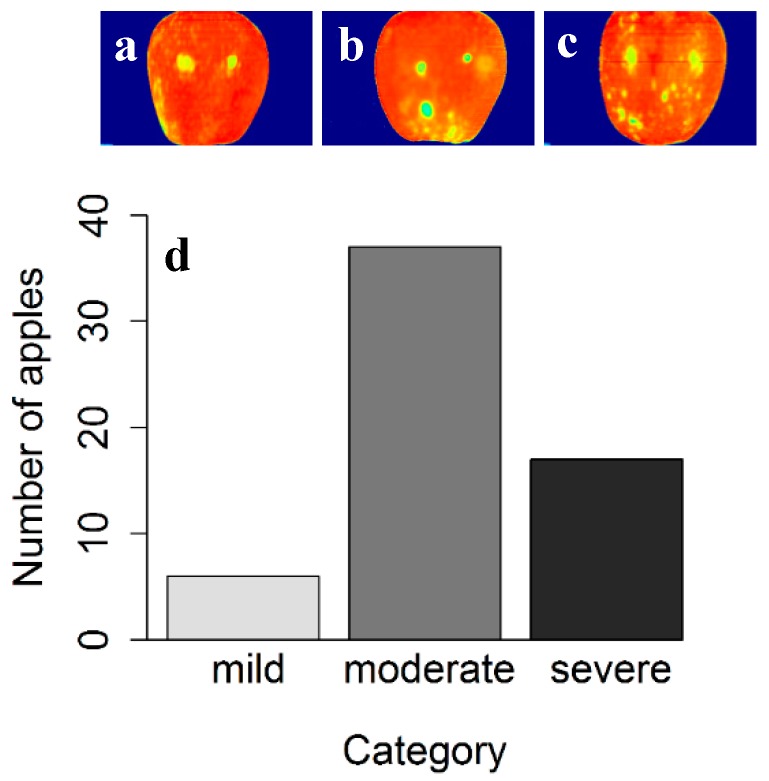
Distribution of bitter pit area in apples. (**a**) mild; (**b**) moderate; and (**c**) severe bitter pit apples, and (**d**) number of apples in each category. The fruit surface includes two saturated reflectance points resulting from light reflection.

Figure 5 shows sample apples with different bitter pit intensities and number of bitter pit apples in three bitter pit apple categories. This result helps in defining the extent of bitter pit disorder on each apple in order to make management decisions in the sorting facilities by categorizing severely, moderately and mildly bitter pitted apples. The mildly and moderately bitter pit apples were separated according to the threshold obtained in the logistic regression for distinguishing between healthy and bitter pit apples. For categorizing moderate and severe bitter pit, the appearance of the apple was taken into account. This categorization can have a great impact on apple production and wholesale marketing, as it prevents the packaging of fruits with bitter pit disorder and helps decisions about the use of such apples in processing rather than the fresh market. The proposed algorithm enables the industry to implement this method for other injuries as well as bitter pit on different apple varieties. This proves that having spatial information in hyperspectral imaging can help in food inspection applications.

## 4. Conclusions

Apple bitter pit detection in early to non-symptomatic stages can prevent packaging of affected produce, thereby reducing associated labor and transportation costs. Based on the spectral dataset analysis of the four seasons, it can be concluded that the spectral bands of 730, 980, 1135, 1250 and 1405 nm have the potential to detect bitter pit. The PLSR and KNN classifiers confirmed the validity of the above bands with accuracies in the ranges of 71–100%. The hyperspectral imaging system of the produce samples had four additional spectral bands (665, 731, 797, 1217, 1283, 1349, and 1410 nm) that can be applied to bitter pit detection. These salient spectral features based on both the hyperspectral spectrometry and imaging techniques can be integrated with imaging tools on the packaging lines in order to sort fruits.

## Figures and Tables

**Table 1:** Details of fruit samples used for spectral evaluation in this study.

Year of Data Collection	Produce Type	Location (in WA State)	Number of Samples	
			Healthy	Bitter Pit
2017	From storage facility *	Bergnoff	30	30
		Horsey Jump	30	30
2016	63 days after field harvest	Prescott	30	30
		Burbank	30	30
2015	63 days after field harvest	Prescott	30	30
		Quincy	30	30
2014	63 days after field harvest	Prescott	20	20
		Burbank	20	20

* 2016 produce was stored in controlled atmospheric conditions by a commercial facility for 5 months prior to sampling for analysis.

**Table 2:** Partial least square regression (PLSR) and k-nearest neighbor (KNN)-derived percent classification accuracies for visible–near-infrared spectrometry (730, 980, 1135, 1250, and 1450 nm), and hyperspectral imaging (665, 731, 797, 1217, 1283, 1349, and 1410 nm) data derived selected features.

Spectral Features (nm)	Year of Data Collection	Classification Accuracy (Average ± Standard Deviation, %)					
			PLSR			KNN	
		Overall Accuracy	False Negative	False Positive	Overall Accuracy	False Negative	False Positive
730, 980, 1135, 1250, 1450	2017	90 ± 3	10 ± 3	0 ± 0	97 ± 0	0 ± 0	3 ± 0
	2016	97 ± 0	3 ± 0	0 ± 0	100 ± 0	0 ± 0	0 ± 0
	2015	76 ± 6	7 ± 4	17 ± 7	71 ± 2	17 ± 4	12 ± 2
	2014	75 ± 5	0 ± 0	25 ± 5	75 ± 10	8 ± 6	17 ± 6
665, 731, 797, 1217, 1283, 1349, 1410	2017	90 ± 0	12 ± 3	2 ± 3	88 ± 9	8 ± 9	9 ± 5
	2016	100 ± 0	0 ± 0	0 ± 0	100 ± 0	0 ± 0	0 ± 0
	2015	93 ± 4	2 ± 3	6 ± 7	67 ± 9	15 ± 9	18 ± 12
	2014	92 ± 3	3 ± 3	11 ± 10	80 ± 5	5 ± 5	14 ± 8

**Table 3:** Classification accuracy using the area threshold estimated using hyperspectral feature analysis.

Number of Spectral Features	Overall Accuracy (%)	False Negative (%)	False Positive (%)
7 features	85.0	5.0	10.0
3 features (665, 731, 797 nm)	82.0	6.0	12.5
4 features (1217, 1283, 1349, 1410 nm)	81.0	3.0	16.0
